# Methyl benzoate exhibits insecticidal and repellent activities against *Bemisia tabaci* (Gennadius) (Hemiptera: Aleyrodidae)

**DOI:** 10.1371/journal.pone.0208552

**Published:** 2018-12-04

**Authors:** Mohammad Munir Mostafiz, Pijush Kanti Jhan, Jae-Kyoung Shim, Kyeong-Yeoll Lee

**Affiliations:** 1 Division of Applied Biosciences, College of Agriculture and Life Sciences, Kyungpook National University, Daegu, Republic of Korea; 2 Institute of Plant Medicine, Kyungpook National University, Daegu, Republic of Korea; 3 Institute of Agricultural Science and Technology, Kyungpook National University, Daegu, Republic of Korea; 4 Sustainable Agriculture Research Center, Kyungpook National University, Gunwi, Republic of Korea; Chinese Academy of Agricultural Sciences, CHINA

## Abstract

Methyl benzoate (MB) is a plant-derived volatile organic compound with insecticidal properties, but such activity has not been evaluated against the sweetpotato whitefly *Bemisia tabaci* (Gennadius) (Hemiptera: Aleyrodidae), a major crop pest. In this study, we tested methyl benzoate control efficacy on *B*. *tabaci* infecting tomato plants in a greenhouse, specifically measuring contact and fumigant toxicity, as well as repellent activity. For direct spray applications of 0% (control), 0.1%, 0.25%, 0.5%, 1%, 2% MB onto tomato leaves infested with adults of *B*. *tabaci* (< 5-d-old), 2% MB showed the highest corrected mortality (100%) at 24 h post-treatment. For residual toxicity in which the same MB solutions were sprayed onto tomato leaves and allowed to dry for 2 h before < 5-d-old adults were released, the 2% MB also showed the highest corrected mortality (100%) at 48 h post-treatment. The lethal median concentration (LC_50_) for eggs, fourth-instar nymphs, and adults were 0.3%, 0.2%, and 0.2%, respectively. In pot culture experiments, 1% MB concentration was found more effective at killing nymphs and preventing adult eclosion than all other concentrations, and gave 100 percent population reduction compared with the control. MB repelled adult whiteflies and caused 96.5% fumigant toxicity within 10 h post-treatment. Repellency and anti-oviposition rates against *B*. *tabaci* had median effective doses of 0.24% and 0.16%, respectively. Our results suggest that MB has strong potential as an environmentally friendly biopesticide for control of *B*. *tabaci* but field trials and further greenhouse studies are required to establish efficacy under more natural conditions.

## Introduction

The sweetpotato whitefly *Bemisia tabaci* (Gennadius) (Hemiptera: Aleyrodidae) is a major pest that hampers productivity of numerous agricultural and horticultural crops worldwide [1,2]. *Bemisia tabaci* has a wide genetic diversity and actually forms a taxonomic complex containing at least 40 cryptic species, including the highly invasive Middle East Asian Minor 1 (MEAM1; formerly B-biotype) and Mediterranean (MED; formerly Q-biotype) groups found in many different countries [3,4,5] and infesting over 900 cultivated/wild plants [6]. MEAM1 and MED transmit over 110 plant pathogenic viruses, most notably the highly destructive tomato yellow leaf curl virus (TYLCV) [7,8,9]. This combination of traits (high genetic diversity, wide host range, invasiveness) makes *B*. *tabaci* especially difficult to manage.

For the past two decades, pesticide use has been common for management of major insect pests, including whiteflies, causing the development of high level of resistance [10,11] even against relatively new insecticide classes (e.g., imidacloprid) [10,12,13]. Complicating this issue is the fact that many countries have banned certain insecticides due to health or environmental reasons. Thus, considerable pressure exists to develop alternatives that are effective against pests, but environmentally friendly and harmless to non-target organisms.

Plant-derived insecticides [14] are more desirable than their synthetic counterparts because the former exhibit rapid environmental biodegradation, multiple modes of action, synergistic effects among constituents, and lower toxicity to non-pests [15–20]. Increasing the use of these more environmentally friendly alternatives can reduce the damage to ecosystems and human health caused by over-application of synthetic insecticides. Successful examples of plant-derived pesticides include pyrethrin from *Chrysanthemum* sp. (Asteraceae), neem from *Azadirachta indica* (Meliaceae), nicotine, rotenoid, and several essential oils (EOs) [21–26]. The latter group contains volatile aromatic compounds that give rise to characteristic plant flavors and fragrances. These aromatics are grouped into three categories: monoterpenes, sesquiterpenes, and aliphatic compounds (alkanes, alkenes, ketones, aldehydes, acids, alcohols) [27,28]. In addition to their effectiveness against pests compared with synthetic pesticides [25,29,30], the high volatility of EOs make them important fumigants against insect pests [30]. Notably, the aromatic ester methyl benzoate (MB, C_6_H_5_CO_2_CH_3_), first extracted from fermented apple juice, has recently been shown to exhibit potent toxicity against various insect species [31]. Like other aromatics, MB occurs as an aroma and odor of many plants [32], including flowers [33,34], and plays significant roles in plant communication with the adjacent environment.

However, there are no data available to show whether MB is effective against *B*. *tabaci*. Therefore, in this study, we carried out various experiments to analyze the effects of MB on the insect pest, including contact and fumigant toxicity, repellency, and control efficacy. Our results successfully demonstrated that MB is a viable biopesticide against *B*. *tabaci*.

## Materials and methods

### Insects and reagents

Since 2009, a colony of *Bemisia tabaci* MED has been maintained in an insect-proof cage (45 × 60 × 90 cm) and fed on suhgwang cultivar of tomato plants (*Lycopersicon lycopersicum*), grown in 1.5-L pots (one plant/pot) filled with potting mix. Growing conditions were 25 ± 1°C, 60 ± 10% relative humidity (RH), and a 16L:8D photoperiod. Our colony is a Mediterranean species (MED; Q biotype) of *B*. *tabaci* species complex. More specifically, our colony belongs to Q1 biotype (mt COI Genbank accession number HM488315) among at least five subgroups of Q biotype [35,36]. The genetic identity of the whitefly colony maintained in the laboratory is checked every six months according to a published protocol [37]. Prior to this experiment, we rechecked with mitochondrial COI gene for conformity.

Methyl benzoate (M29908-500G), Tween 20 (P1379-25ML), and Tween 80 (P1754-25ML) were purchased from Sigma-Aldrich (St. Louis, MO, USA). Methyl benzoate solutions (0.1%, 0.25%, 0.5%, 1%, 2%) were prepared with distilled water containing 1% emulsifier (v/v) consisting of a 1:1 ratio of Tween 20 and Tween 80. For controls, distilled water containing 0.5% Tween 20 (v/v) and 0.5% Tween 80 (v/v) were used.

### Contact toxicity of MB against adults of *B*. *tabaci*

**Fig 1:**
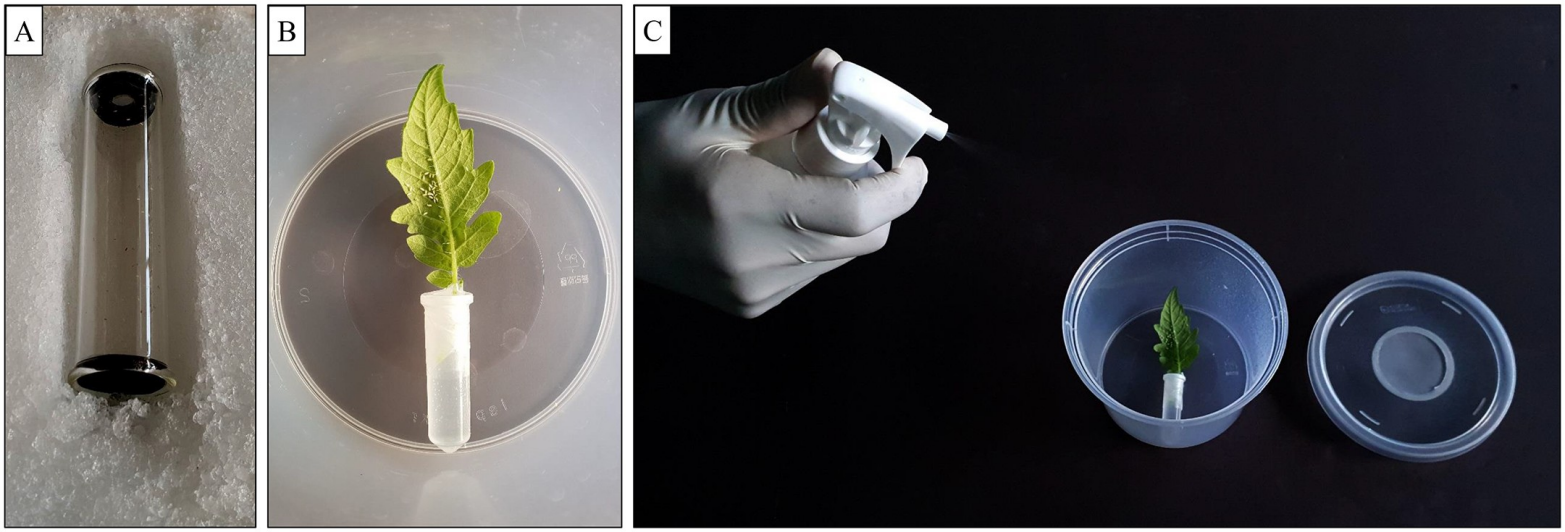
Experimental setup for the direct spray application of MB solutions onto the whitefly-infested tomato leaves. (A) Adults of *Bemisia tabaci* (less than 5 days old) from the rearing colony were collected in a group and placed on ice; (B) Batches of 25 cold-immobilized adults were placed on test leaves in breeding cups; (C) MB solutions were sprayed onto each leaf using a glass spray bottle.

Two methods were employed to evaluate MB contact toxicity. First, MB solutions were sprayed directly onto *B*. *tabaci*-infested tomato leaves. The bioassay was conducted using an insect breeding cup (12 cm diameter × 8 cm height) with aeration via a ventilation hole in the lid that was covered by a nylon mesh. To prepare a test arena, a tomato leaf petiole was inserted into a 1.5 mL Eppendorf plastic tube filled with water and placed in each breeding cup with the lower leaf surface facing upward (Fig 1). Adults of *B*. *tabaci* (less than 5 days-old) from the rearing colony were collected in a group and placed on ice. Batches of 25 cold-immobilized adults were placed on test leaves in breeding cups with a fine brush and allowed to warm and regain mobility. When 20 adults had recovered from cold immobilization and had resumed activity, ~0.5 mL MB solutions (0%, 0.1%, 0.25%, 0.5%, 1% and 2%) were sprayed onto each leaf using a glass spray bottle. After air-drying, each breeding cup was covered with a lid. Control leaves were sprayed only with ~0.5 mL of distilled water solution (see under “Insects and reagents”). Mortality was measured at 6 h intervals for 3 d, via a lack of response when probed with a small brush.

Second, MB solutions were sprayed onto leaves first before releasing adults. Tomato leaf petioles were prepared as described above, followed by spray application of MB solutions (~0.5 mL total). After air-drying for 2 h, 20 adults (< 5-d-old) were transferred onto treated leaves. Control leaves were again treated with only distilled water solution. Mortality was measured at 12 h intervals for 120 h post-treatment. Both experiments were replicated three times.

### Contact toxicity of MB against *B*. *tabaci* during egg and nymphal stages

Adults of *B*. *tabaci* were allowed to lay eggs on fresh tomato plants for 24 h. Seven-day-old eggs and 4-d-old fourth-instar nymphs were then collected to be used in MB contact toxicity experiments.

Eggs were first counted under a dissection microscope (Olympus, Tokyo, Japan). If the leaves had more than 30 eggs, the excess were destroyed with a needle and discarded. These leaves were then dipped for 5 s in MB solutions of five different concentrations (0.1%, 0.25%, 0.5%, 1%, and 2%) plus the control solution, followed by air-drying for 30 min [38,39]. To keep eggs moist, leaves were placed individually on a bed of 1% agar (Junsei Chemical Co., Ltd., Tokyo, Japan) in petri dishes (5 cm diameter × 1.5 cm height). Unhatched eggs and newly emerged nymphs were counted daily for 6 d post-application. The unhatched eggs, along with any individuals unable to emerge from egg shells, were considered dead. Ovicidal effects were calculated as the percentage of eggs hatched in each MB treatment.

To determine nymphicidal effects, leaves with fourth-instar nymphs (n = 15) were dipped and dried as described for eggs. Adult eclosion rate was determined daily until 6 d post-application. Nymphs were considered dead if they were shriveled, discolored, or dried out. Both experiments were replicated three times.

### Control efficacy of MB spray on tomato plants

To determine control efficacy of *B*. *tabaci* on tomato plants, MB solutions were sprayed onto tomato plants infested with whitefly nymphs. At first, the number of nymphs in each leaf was counted by viewing them using a 10× Loupe (Nikon, Tokyo, Japan). Three sprays were conducted on the potted tomato plants: the first spray was done after the nymphs were counted, the second spray 7 days after the first spray and the third spray 7 days after second spray. Leaves were observed weekly under the 10× Loupe to determine nymphal mortality and adult eclosion rates. Nymphs were considered dead if they were shriveled, discolored or dried out. The presence of exuviae from fourth-instar nymphs was an indicator of successful eclosion. Each test was replicated three times.

### Fumigant toxicity of MB against adults of *B*. *tabaci*

Filter papers (2.5 cm diameter; Whatman No. 1, Maidstone, England) were first wetted uniformly with applications of 100 μL MB solutions (0% [control], 0.1%, 0.25%, 0.5%, and 1%). Once dried, filter papers were attached to the bottom of a glass cylinder (15 cm height × 1.5 cm diameter) that was split into two chambers with a mesh net ring (1.5 cm diameter). Adults of *B*. *tabaci* (n = 30) were released in the upper chamber to prevent direct contact with the treated filter paper, and the cylinder was sealed with Parafilm (Bemis Company Inc., Neenah, USA). Mortality rates were recorded 2, 4, 6, 8, and 10 h post-exposure. Three replications were performed for each MB concentration and for the control.

### Methyl benzoate repellency against adults of *B*. *tabaci*

**Fig 2:**
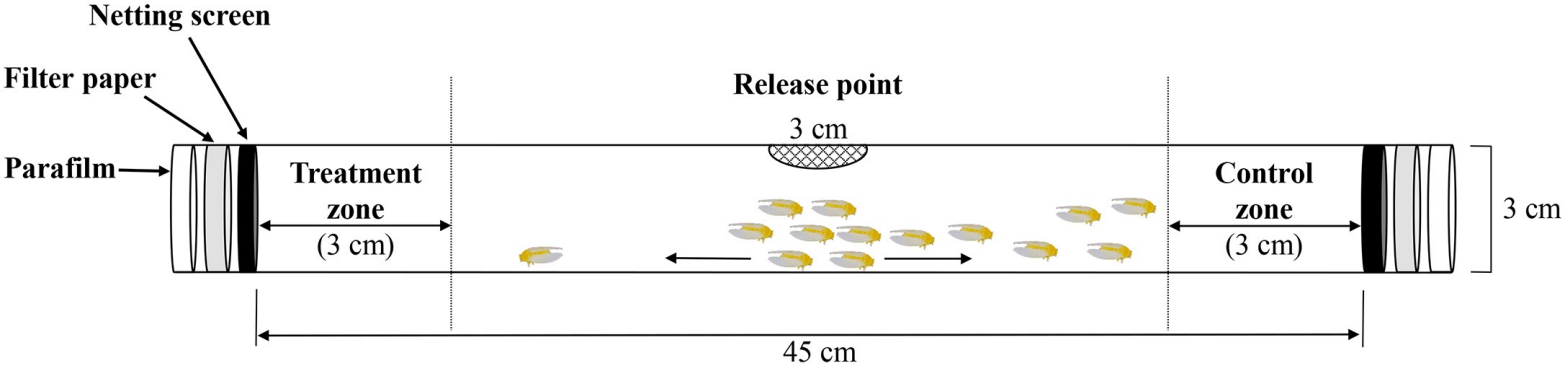
The apparatus used in bioassays. Before the assay, methyl benzoate (treatment) solution was applied to the filter paper and placed between the netting screen and the bottom of the assembly, and then the tube was covered with parafilm. The opposite end of the tube was used as the control.

A bioassay tube [40] was constructed of clear plastic piping (45 cm length × 3 cm in diameter) with two open ends and a hole in midway down the tube (Fig 2). Small chambers on both ends were formed with the insertion of mesh net rings. One end was left empty as the control, while MB-treated filter paper (100 μL each of 0.1%, 0.25%, 0.5%, 1%, and 2% solutions) was placed in the other end. Randomly collected adults (n = 20) were released into the pipe through the middle hole. At 1 h, 3 h, and 6 h post-release, adults in the treatment and control zones were counted. Each test was replicated three times. The tube was cleaned with soap and water, rinsed with 70% ethanol, and then dried between treatments.

### Repellency and anti-oviposition activity (deterrence rate) of tomato plants treated with MB

Tomato plants grown in small pots (9 cm height × 9 cm diameter) were sprayed with 10 mL of five MB solutions (0.1%, 0.25%, 0.5%, 1%, or 2%) or control solution. Two hours later, one treated and one control plant (separated by 30 cm) were placed within a small mesh-covered cage (50 × 50 × 40 cm). Randomly selected adults (n = 100) were then released, equidistant between the two plants. After 24 and 48 h of exposure, adults per plant were counted in the early morning when the insects were inactive. Half of the leaves per treatment were collected at 24 h post-treatment time (PTT), while the remainder was collected at 48 h PTT. Eggs on treatment and control plants were counted under a dissection microscope. Each test was replicated three times.

### Statistical analysis

One-way ANOVA, followed by a post-hoc Tukey’s HSD test, was used to determine differences in toxicity, repellency, and oviposition percentages (*P* < 0.05) [41]. All percentage mortality data were corrected using Abbott’s formula [42]. Log-probit regression was used to calculate lethal median time (LT_50_) based on corrected mortality from various MB concentrations [41]. Corrected mortality data also were used to determine lethal median concentration (LC_50_) [41]. Significant between-treatment differences were determined using 95% confidence intervals (CI). Adult repellency and oviposition percentage was calculated using the formula PR (%) = [(C-T)/(C+T)] × 100 [43]. All analyses were performed in SAS version 9.4 [41]. All the graphs were drawn with SigmaPlot 12.5 [44].

## Results

### Contact toxicity of MB against adults of *B*. *tabaci*

**Fig 3:**
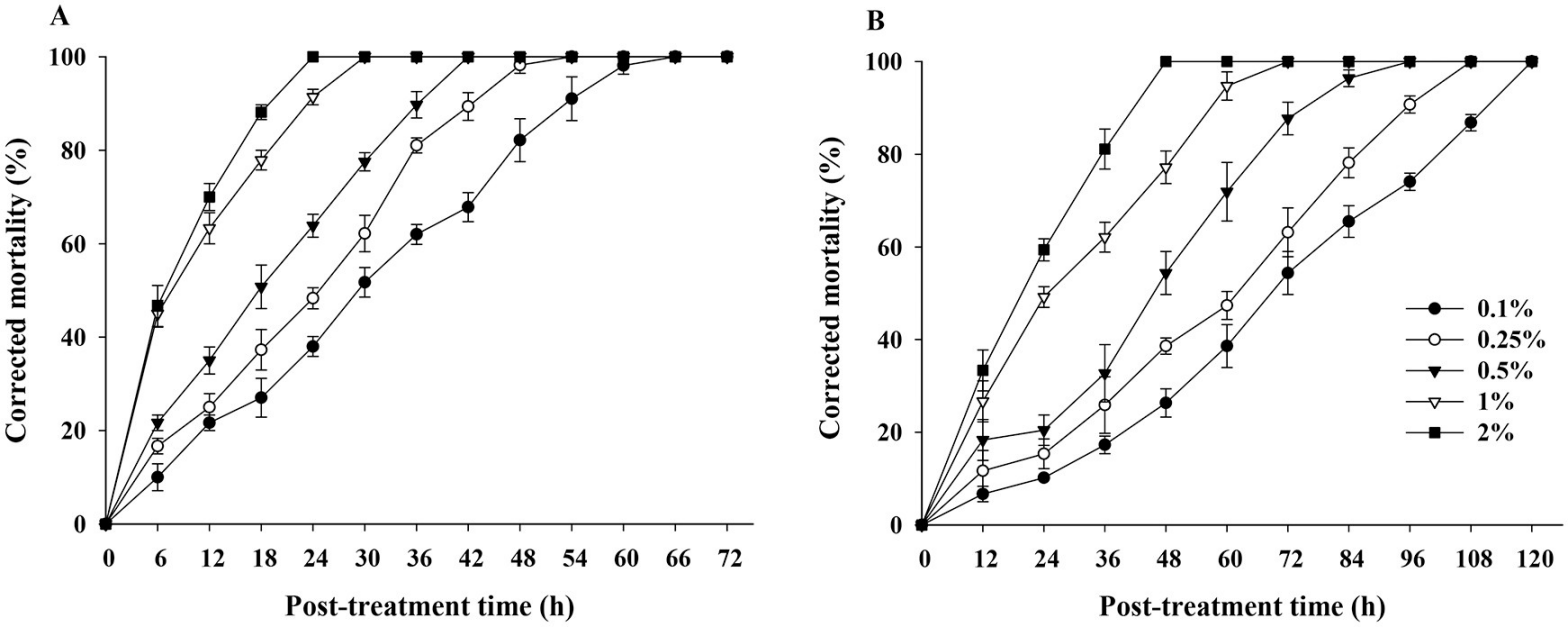
Contact toxicity of methyl benzoate against adults of *Bemisia tabaci*. For direct spray application on whitefly infested tomato leaves (A), various concentrations of MB solution (~0.5 mL total) were sprayed on < 5-d-old adults of *B*. *tabaci* (n = 20). For residual toxicity (B), various concentrations of MB solution (~0.5 mL total) were first sprayed on tomato leaves and allowed to dry for 2 h before < 5-d-old adults (n = 20) were released into an insect breeding cup containing the leaves. Values are the means of three replicates.

Direct spray application of MB significantly increased adult mortality on whitefly infested tomato leaves by 12 h (*N* = 3, *F* = 63.82; df = 4, 14; *P* < 0.0001) and 24 h (*N* = 3, *F* = 196.44; df = 4, 14; *P* < 0.0001) (Fig 3A). Mortality increased over time and decreased gradually from higher to lower MB concentrations (Fig 3). Corrected mortality was 100% after 24 h PTT for 2% MB, 30 h PTT for 1%, 42 h PTT for 0.5%, 54 h PTT for 0.25%, and 66 h PTT for 0.1% (Fig 3A). In contrast, corrected mortality in residual assays was 100% after 48 h PTT for 2% and 72 h PTT for 1%. A low of 54.4% corrected mortality was recorded for 0.1% MB after 72 h PTT (Fig 3B). Mortality rates at 12 h (*N* = 3, *F* = 11.64; df = 4, 14; *P* = 0.005) and 24 h (*N* = 3, *F* = 76.06; df = 4, 14; *P* < 0.0001) were significantly different at all MB concentrations (Fig 3B).

LT_50_ values were calculated after 72 h and 120 h PTT for direct spray application on whitefly infested tomato leaves and residual assays of MB (Tables 1 and 2). The LT_50_ values of 2% and 1% MB under direct spray application were 7.1 h (*χ*^*2*^ = 15.8, df = 10, *P* = 0.106) and 8.1 h (*χ*^*2*^ = 24.2, df = 10, *P* = 0.007), respectively (Table 1). In the residual assay, the LT_50_ values of 2% and 1% MB were 17.8 h (*χ*^*2*^ = 23.1, df = 8, *P* = 0.003) and 22.8 h (*χ*^*2*^ = 36.1, df = 8, *P* < 0.0001), respectively (Table 2). Finally, LC_50_ for adults of *B*. *tabaci* were 0.2% (v/v) MB for direct spray application assay and 0.3% (v/v) MB for the residual assay (Table 3).

**Table 1:** Comparing LT_50_ of methyl benzoate (MB) against *Bemisia tabaci* adults after direct spray application of different concentrations (n = 60).

MB concentration (%)	LT_50_ (h)	95% CI^+^ (lower-upper)	Slope (± SE)	χ^2^ (df)
2.0	7.1a	(6.2–8.1)	3.6 (0.3)	15.8 (10)
1.0	8.1ab	(6.1–9.6)	3.2 (0.4)	24.2 (10)
0.5	14.6b	(11.3–17.8)	3.4 (0.4)	55.7 (10)
0.25	18.7c	(14.0–23.1)	3.5 (0.5)	83.2 (10)
0.1	24.3d	(19.1–29.2)	3.3 (0.5)	72.5 (10)

^+^Confidence interval

The LT_50_ value was calculated using corrected mortality. LT_50_ values followed by different letters are significantly different (95% CI) across MB concentrations

**Table 2:** Comparing LT_50_ against *Bemisia tabaci* adults, treated with different methyl benzoate (MB) concentrations in a residual assay (n = 60).

MB concentration (%)	LT_50_ (h)	95% CI^+^ (lower- upper)	Slope (± SE)	χ^2^ (df)
2.0	17.8a	(14.1–21.1)	4.1 (0.5)	23.1 (8)
1.0	22.8ab	(17.2–28.1)	3.5 (0.4)	36.1 (8)
0.5	35.7bc	(24.8–46.1)	3.6 (0.7)	89.5 (8)
0.25	48.0cd	(34.3–61.8)	3.4 (0.7)	93.2 (8)
0.1	60.5d	(47.0–76.7)	3.4 (0.6)	77.1 (8)

^+^Confidence interval

The LT_50_ value was calculated using corrected mortality. LT_50_ values followed by different letters are significantly different (95% CI) across MB concentrations

**Table 3:** Comparative lethal median concentration (LC_50_) values of methyl benzoate (MB) for *Bemisia tabaci* eggs, nymphs, and adults.

Insect stage	LC_50_ (%, v/v)	95% CI^+^ (lower- upper)	Slope (± SE)	χ^2^ (df)
LC_50_ 144 h after exposure				
Eggs	0.3	(0.2–0.6)	1.6 (0.3)	10.5 (3)
LC_50_ 144 h after exposure				
Nymphs	0.2	(0.1–0.3)	2.1 (0.3)	8.8 (3)
LC_50_ 24 h after exposure (direct spray application assay)				
Adults	0.2	(0.0–0.4)	1.8 (0.4)	17.9 (3)
LC_50_ 48 h after exposure (residual assay)				
Adults	0.3	(0.1–0.6)	1.8 (0.4)	17.9 (3)
LC_50_ 10 h after exposure (fumigant toxicity)				
Adults	0.2	n/a	1.7 (0.6)	17.7 (2)

^+^Confidence interval

The LC_50_ value was calculated using corrected mortality. n/a, no confidence interval observed and therefore no probit analysis performed

### Contact toxicity of MB against *B*. *tabaci* eggs and fourth-instar nymphs

**Fig 4:**
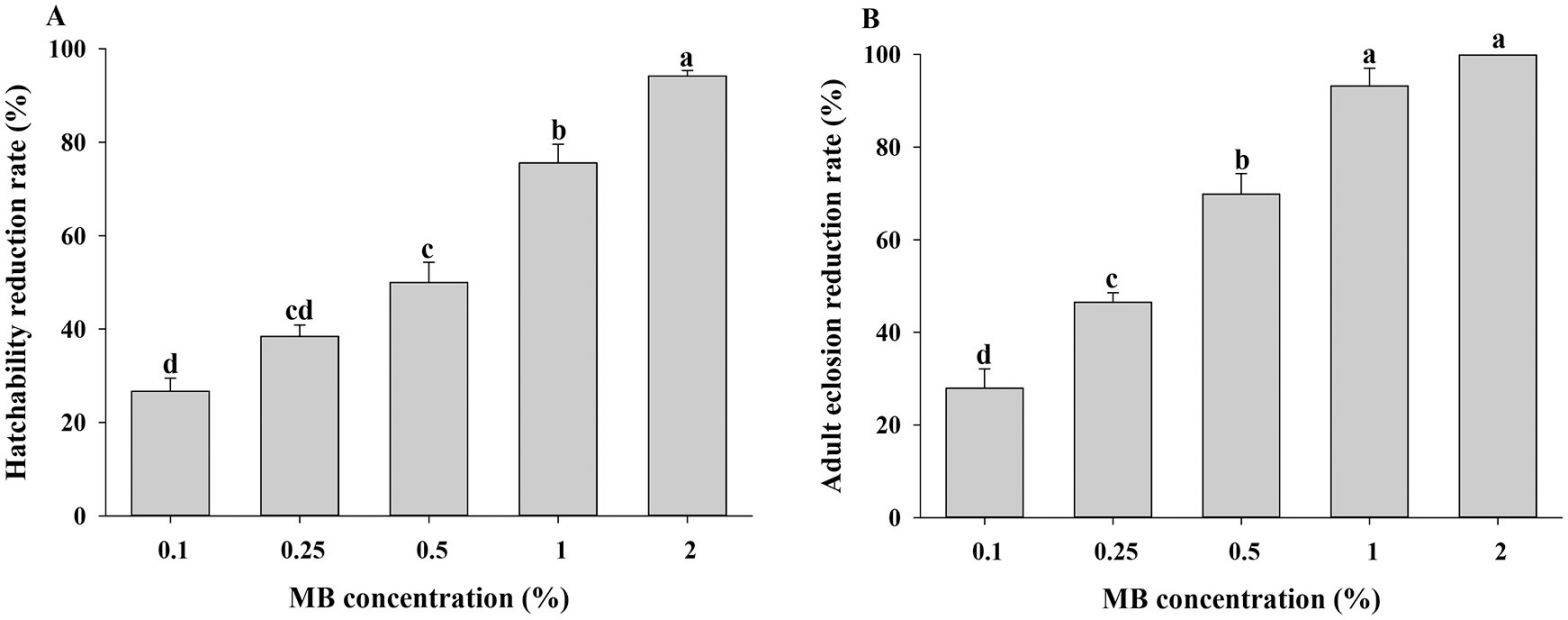
Contact toxicity of methyl benzoate against *Bemisia tabaci* eggs and fourth-instar nymphs. Tomato leaves infested with 7-d-old eggs (n = 30) or 4-d-old fourth-instar nymphs (n = 15) were dipped into the various concentrations of MB solutions. Egg hatch reduction rates (A) and adult eclosion reduction rates (B) were assessed at 6 d post-treatment. Values are means of three replications. Means denoted by the same letters are not significantly different at α = 0.05 (*N* = 3, df = 4, 14; for ovicidal effects, *F* = 75.82; *P* < 0.0001; for nymphicidal effects, *F* = 83.14; *P* < 0.0001).

Egg hatch reduction values were 26.7%, 38.4%, 50.1%, 75.6%, and 94.2% under the leaf dipping method at concentrations of 0.1%, 0.25%, 0.5%, 1% and 2% MB, respectively (Fig 4A). Egg hatch rate was significantly inhibited in a concentration-dependent manner (*N* = 3, *F* = 75.82; df = 4, 14; *P* < 0.0001) (Fig 4A). Adult eclosion reduction rates by the leaf dipping method with 0.1, 0.25, 0.50, 1 and 2% MB concentrations were 27.9%, 46.5%, 69.8%, 93.2% and 100%, respectively (Fig 4B). Adult eclosion rate was significantly inhibited in a concentration-dependent manner (*N* = 3, *F* = 83.14; df = 4, 14; *P* < 0.0001) (Fig 4B). The LC_50_ value for egg mortality was 0.3% (v/v) MB and for the mortality of fourth-instar nymphs was 0.2% (v/v) MB (Table 3).

### Control efficacy of methyl benzoate spray on tomato plants

We observed a significant negative effect of 0.2%, 0.5%, and 1% MB sprays on adult eclosion rates (*N* = 3, *F* = 10.85; df = 3, 11; *P* = 0.0034, Table 4). At 7 d after final spraying, 0–12 adults were present per plant, compared with over 50 adults in the control condition. Eclosion was completely eliminated under 1% MB, but 0.5% and 0.2% MB also were effective in reducing eclosion.

**Table 4:** Efficacy of methyl benzoate against *Bemisia tabaci* in pot culture.

MB concentration (%)	Mean number of nymphs/plant before spraying	Mean number of *B*. *tabaci* alive per treatment	Population reduction over control (%)
		After 3 sprays	
1.0	26.7	0.0 ± 0.0b	100
0.5	42.0	6.7 ± 0.9b	87.1
0.2	46.7	12.0 ± 3.2b	76.8
Control	58.3	51.7 ± 13.7a	-

Data are presented as means ± SE (n = 3). Means followed by same letters are not significantly different within a single column, based on one-way ANOVA and Tukey’s HSD post-hoc test (*P* < 0.05).

### Fumigant toxicity of methyl benzoate against adults of *B*. *tabaci*

Maximum fumigant toxicity occurred at 1% MB at 10 h PTT (*N* = 3, *F* = 149.3; df = 3, 11; *P* < 0.0001, Table 5). Toxicity increased over time, and corrected mortality rates were highest at 10 h PTT, reaching 37.2%, 45.3%, 66.3%, and 96.5% for 0.1%, 0.25%, 0.5%, and 1% MB, respectively (Table 5). The lethal median time (LT_50_) was 5.0 h (*χ*^*2*^ = 26.1, df = 3, *P* < 0.0001) for 1% MB concentration, significantly different from the other MB concentrations (Table 6).

**Table 5:** Fumigant toxicity of methyl benzoate determined as a percentage of *Bemisia tabaci* corrected mortality after 2 h, 4 h, 6 h, 8 h, and 10 h exposure in the laboratory.

MB concentration (%)	Corrected mortality (%)				
	2 h	4 h	6 h	8 h	10 h
1.0	15.6 ± 1.1a	24.4 ± 2.9a	55.1 ± 1.8a	72.4 ± 1.4a	96.5 ± 1.2a
0.5	10.0 ± 1.9ab	21.0 ± 2.2a	40.5 ± 2.4b	55.2 ± 0.6b	66.3 ± 1.9b
0.25	7.8 ± 1.1b	16.7 ± 1.9ab	27.1 ± 1.9c	42.5 ± 1.3c	45.3 ± 1.6c
0.1	4.4 ± 1.1b	10.0 ± 1.9b	18.1 ± 2.9c	24.1± 2.5d	37.2 ± 1.7c

Data are presented as means ± SE (n = 3). Means followed by same letters within a column are not significantly different, based on one-way ANOVA and Tukey’s HSD post-hoc test (*P* < 0.05).

**Table 6:** LT_50_ values from fumigation bioassay of methyl benzoate (MB) against *Bemisia tabaci* adults for different concentrations (n = 90).

MB concentration (%)	LT_50_ (h)	95% CI^+^ (lower- upper)	Slope (± SE)	χ^2^ (df)
1.0	5.0a	(2.4–8.5)	3.5 (0.9)	26.1 (3)
0.5	7.2b	(6.5–8.2)	2.6 (0.3)	2.1 (3)
0.25	11.2c	(9.3–15.1)	2.0 (0.3)	1.4 (3)
0.1	16.3d	(12.5–26.8)	2.0 (0.3)	1.5 (3)

^+^Confidence interval.

The LT_50_ value was calculated using corrected mortality. LT_50_ values followed by different letters are significantly different (95% CI) across MB concentrations

### Repellency of methyl benzoate to *Bemisia tabaci* adults

**Fig 5:**
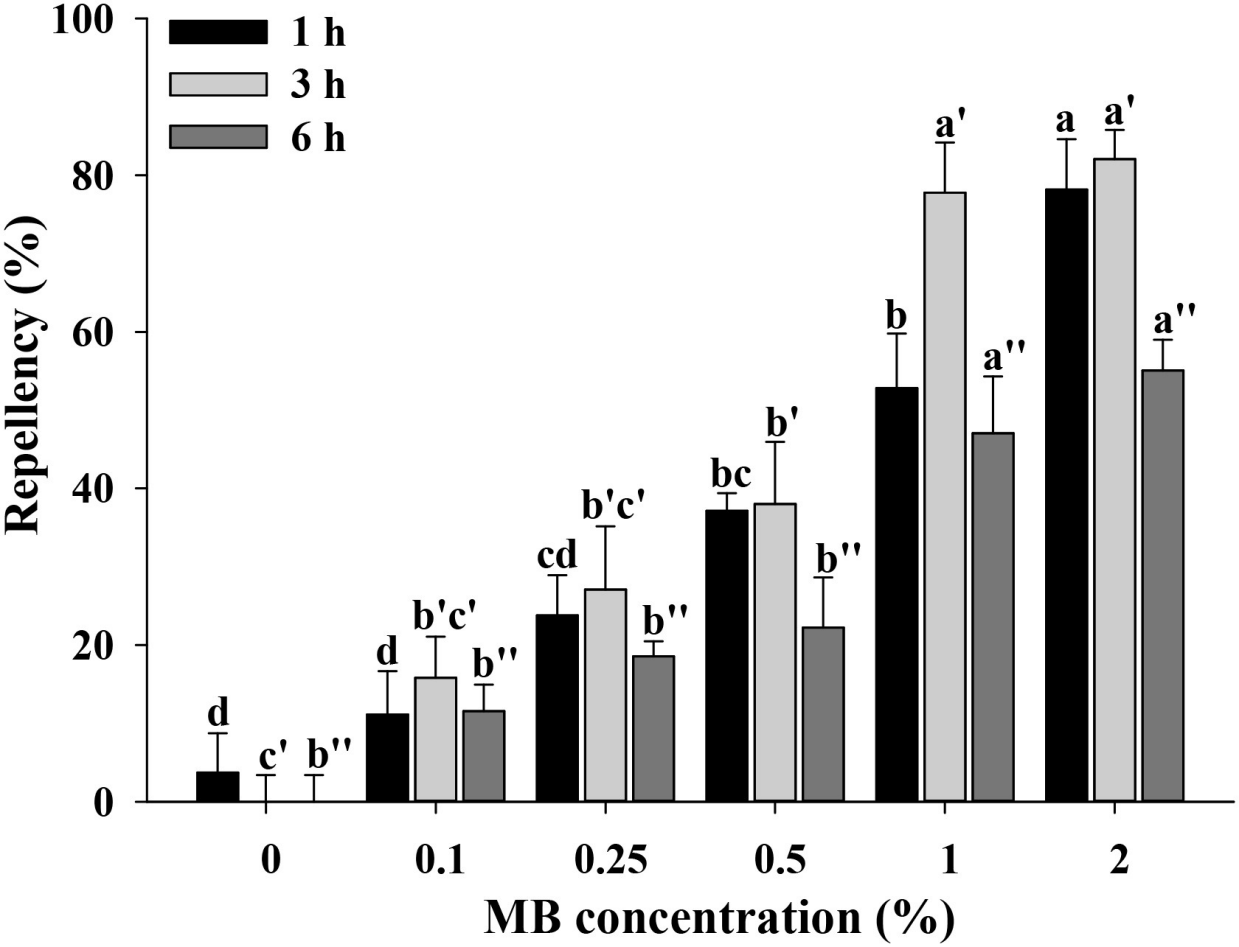
Repellency effects of methyl benzoate against adults of *Bemisia tabaci*. A binary-choice bioassay tube was used to determine behavioral responses of *B*. *tabaci* after encountering 0% (control), 0.1%, 0.25%, 0.5%, 1%, and 2% MB solutions. Repellency was determined at 1, 3, and 6 h post-treatment. Values are the means of three replications. Means followed by different superscript letters are significantly different at α = 0.05 (*N* = 3, df = 5, 17; for 1 h, *F* = 26.11; *P* < 0.0001; for 3 h, *F* = 29.99; *P* < 0.0001; for 6 h, *F* = 19.87; *P* < 0.0001).

The ability of MB to repel *B*. *tabaci* was concentration and time-dependent. All MB concentrations resulted in significant repellency after 1 h (*N* = 3, *F* = 26.11; df = 5, 17; *P* < 0.0001), 3 h (*N* = 3, *F* = 29.99; df = 5, 17; *P* < 0.0001), and 6 h (*N* = 3, *F* = 19.87; df = 5, 17; *P* < 0.0001) of the bioassay (Fig 5). Repellency was highest under 2% MB at 1, 3, and 6 h PTT, with means of 78.2 ± 6.4, 82.1 ± 3.7, and 55.1 ± 3.9%, respectively (Fig 5). After 6 h, however, repellency decreased over time for all concentrations (0.1%, 0.25%, 0.5%, 1%, and 2%). At 6 h PTT, repellency of 0.5%, 0.25% and 0.1% MB was identical to that of the control (0% MB) (Fig 5).

### Repellency and anti-oviposition activities of MB sprayed tomato plants

**Fig 6:**
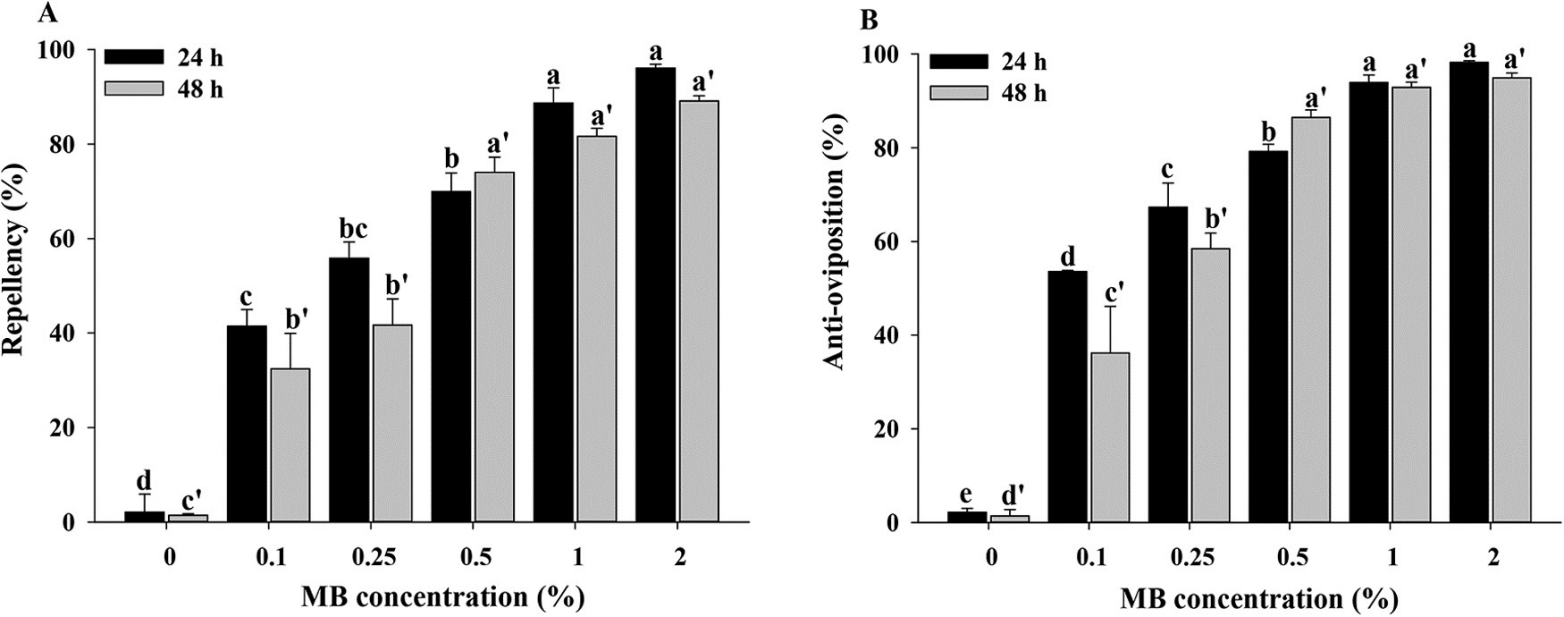
Repellency and anti-oviposition activity of tomato plants sprayed with methyl benzoate. Tomato plants were treated with 0%, 0.1%, 0.25%, 0.5%, 1%, and 2% MB solutions. One non-treated and one treated tomato plant were placed together within a single cage before releasing 100 adults of *B*. *tabaci*. Repellency was determined through counting *B*. *tabaci* on leaves (n = 100) of both plants at 24 and 48 h post-treatment. Eggs on leaves (n = 10) of each plant were counted at 24 and 48 h post-treatment to determine anti-oviposition activity. Values are means of three replications. For (A) repellency, means marked with different superscript letters are significantly different at α = 0.05 (*N* = 3, df = 5, 17; for 24 h, *F* = 108.56; *P* < 0.0001; for 48 h, *F* = 68.20; *P* < 0.0001). For (B) anti-oviposition, means followed by different superscript letters are significantly different at α = 0.05 (*N* = 3, df = 5, 17; for 24 h, *F* = 231.17; *P* < 0.0001; for 48 h, *F* = 71.77; *P* < 0.0001).

Escape from the treated tomato plants increased as MB concentrations rose. Maximum repellency was observed under 2% MB, after 24 h (96.1%) and 48 h (89.1%) of exposure. Repellency differed significantly across all concentrations at 24 h (*N* = 3, *F* = 108.56; df = 5, 17; *P* < 0.0001) and 48 h (*N* = 3, *F* = 68.20; df = 5, 17; *P* < 0.0001) of the bioassay (Fig 6A). Repellency was lowest (41.4% and 32.4%) under 0.1% MB after 24 h and 48 h of exposure, respectively (Fig 6A).

The most effective oviposition deterrent after 24 h (98.2% deterrence) and 48 h (94.9%) of exposure was 2% MB solution (Fig 6B). In contrast, 0.1% MB concentration resulted in deterrence percentages of 53.54% and 36.16% at 24 h and 48 h of exposure (Fig 6B). Oviposition deterrence differed significantly between all concentrations at 24 h (*N* = 3, *F* = 231.17; df = 5, 17; *P* < 0.0001) and 48 h (*N* = 3, *F* = 71.77; df = 5, 17; *P* < 0.0001) of exposure (Fig 6B).

## Discussion

Our study demonstrated that MB had strong lethal and repellent effects against *B*. *tabaci*. We observed contact toxicity among eggs, nymphs, and adults, as well as fumigant toxicity against adults. Binary choice experiments revealed that MB successfully repelled adults, and treating tomato plants with MB significantly reduced attraction to plants and egg-laying of *B*. *tabaci* on the leaves. These findings suggest that MB has multiple effects on physiological and behavioral characteristics during the development of *B*. *tabaci*. However, we did not try the use of MB for control of *B tabaci* in either the open greenhouse conditions (un-caged plants) or in open crop fields, and such studies should be conducted in the future.

Methyl benzoate not only effectively prevented oviposition of *B*. *tabaci* and inhibited subsequent nymphal development, but also caused complete mortality of adults. Under 2% MB, adult mortality completed by 24 h post–spray treatment and 48 h post-residual treatment (Fig 3). Mortality rates of all tested developmental stages of *B*. *tabaci* increased with MB concentration, although LC_50_ generally remained similar during development. These results are consistent with a previous study on *Drosophila suzukii* and *Plutella xylostella*, both demonstrating clear insecticidal activity at 1% MB concentration [31,45]. In addition, the concentration at which MB became effective against *B*. *tabaci* was similar to the performance of other plant volatile compounds; 0.12% citronellol and 0.34% citronellal were toxic against *B*. *tabaci* [46,47].

Methyl benzoate also has strong fumigant toxicity against *B*. *tabaci* adults, with 96.5% mortality within only 10 h of 1% MB treatment (Table 5). In contrast, 24 h of citronellol exposure yielded LC_50_ values of 1.69 μg/cm^3^ against *B*. *tabaci* MED [48]. Thus, MB had faster-acting fumigant toxicity than citronellol.

Binary choice tests and treatment of tomato plants indicated strong repellent activity against *B*. *tabaci* adults. Methyl benzoate treated tomato plants repelled over 80% of *B*. *tabaci* within 48 h. In contrast, limonene treatment resulted in a deterrent effect on adults of *B*. *tabaci* that was 62% higher than the control [49]. Furthermore, MB also lowered egg-laying by over 85%, broadly consistent with the oviposition-deterrent action (80%) of limonene [49] and inhibitory effect (46.1%) of nutmeg (*Myristica fragrances*) essential oil on egg-laying by *B*. *tabaci* after 24 h of exposure [50]. Reduced egg deposition on leaves may be due to the fact that MB residue successfully repelled *B*. *tabaci* females. This possibility has been previously suggested [51], as part of the four mechanisms that inhibit insect behavior: repellency, locomotor stimulation, suppression, and deterrence. An alternative explanation, however, is that MB can directly inhibit the physiological and biochemical mechanisms associated with oviposition. Clearly, we require more research to differentiate between these two hypotheses. Nevertheless, our results suggest that MB can repel adults of *B*. *tabaci* and deter their oviposition, making the compound a promising tool for improving pest control. This suggests that the compound should be tested further to evaluate its potentiality in field applications for control of whiteflies in different crops.

In conclusion, this study is the first to report multiple forms of MB insecticidal activity (toxic, repellent, and oviposition deterrent) against *B*. *tabaci*. Our use of both laboratory and greenhouse experiments enhance the validity of these results. Of course, more studies are necessary to fully understand the specific modes of MB action. Therefore, field evaluation efficacy of MB in open greenhouse and open field experiments to control whiteflies in different crops should be conducted to determine the effective dose that also is non-toxic to field crops for commercial use.

## Supporting information

S1 File Direct spray application, residual, fumigation, repellency and oviposition deterrency of methyl benzoate (MB) on *Bemisia tabaci*. (XLSX)
